# Gencube: centralized retrieval and integration of multi-omics resources from leading databases

**DOI:** 10.1093/bioinformatics/btaf128

**Published:** 2025-04-25

**Authors:** Keun Hong Son, Je-Yoel Cho

**Affiliations:** Department of Biochemistry, College of Veterinary Medicine, Seoul National University, Seoul, 08826, Korea; Comparative Medicine and Disease Research Center (CDRC), Science Research Center (SRC), Seoul National University, Seoul, 08826, Korea; BK21 PLUS Program for Creative Veterinary Science Research and Research Institute for Veterinary Science, Seoul National University, Seoul, 08826, Korea; Department of Biochemistry, College of Veterinary Medicine, Seoul National University, Seoul, 08826, Korea; Comparative Medicine and Disease Research Center (CDRC), Science Research Center (SRC), Seoul National University, Seoul, 08826, Korea; BK21 PLUS Program for Creative Veterinary Science Research and Research Institute for Veterinary Science, Seoul National University, Seoul, 08826, Korea

## Abstract

**Motivation:**

The volume of multi-omics data for diverse species is growing at an unprecedented rate, with new genome assemblies, related annotations, and high-throughput sequencing resources being submitted daily to various genomic data repositories. In response to this data influx, both existing and new databases are establishing optimized hierarchical structures to manage the vast amount of information. However, the lack of accessible command-line tools, combined with the functional limitations and unintuitive design of existing options, presents significant challenges for researchers. This gap underscores a critical need for a tool that enables streamlined retrieval and integration of omics data across these diverse repositories.

**Results:**

We have developed Gencube, a command-line tool that enables centralized retrieval and integration of a comprehensive set of six different data types—genome assemblies, gene sets, annotations, sequences, comparative genomic data, and NGS-based omics resources—from various leading databases.

**Availability and implementation:**

Gencube is a free and open-source tool, with its code available on GitHub: https://github.com/snu-cdrc/gencube and also archived on Zenodo: https://doi.org/10.5281/zenodo.14607649.

## 1 Introduction

The study of genomes has become central to modern biology, driving discoveries in genetics, evolution, and disease mechanisms. By deciphering the information stored in genomes, researchers have been able to identify key regulatory elements, understand species diversity, and develop precision medicine approaches. To further advance these fields, reference-quality genome assemblies across diverse species, comprehensive annotations, and extensive experimental sequencing data—such as bulk and single-cell RNA-seq, ChIP-seq, and ATAC-seq—are essential resources.

Technological breakthroughs, advanced computational methods, and decreasing sequencing costs have expanded the breadth and depth of genome generation efforts. The breadth of these initiatives is evident in ambitious biodiversity projects like the Earth BioGenome Project (EBP) and its affiliates, including the Vertebrate Genomes Project and Zoonomia (Lewin *et al.* 2018, Zoonomia Consortium 2020, Rhie *et al.* 2021). The depth of these efforts is demonstrated by the enhanced understanding of genomic variation within species, as seen in the Human Pangenome Reference Consortium and Dog10K (Liao *et al.* 2023, Meadows *et al.* 2023).

Newly generated genomes have been submitted to GenBank, and traditional databases like RefSeq, UCSC Genome Browser, and Ensembl have been pivotal in providing curated genomes and annotations predicted by various approaches (O'Leary *et al.* 2016, Nassar *et al.* 2023, Martin *et al.* 2023, Sayers *et al.* 2024). However, to quickly provide access to the rapidly increasing genomic resources, new repositories like UCSC GenArk (Clawson *et al.* 2023) and Ensembl Beta (formerly Ensembl Rapid Release) were launched. At the same time, Zoonomia released gene annotations and comparative genomic data inferred using the TOGA (Tool to infer Orthologs from Genome Alignments) method (Kirilenko *et al.* 2023). However, programmatic access to all these new repositories is not available. Additionally, these groups use different chromosome naming conventions, making it difficult to use their resources together. For these reasons, there is a clear need for a solution that enables comprehensive searching across various databases and provides features for data download and integration.

Currently, most high-throughput sequencing data are stored and managed by the International Nucleotide Sequence Database Collaboration (INSDC), which coordinates with SRA, ENA, and DDBJ (Arita *et al.* 2021). The key challenge lies in finding data suitable for specific research and obtaining the corresponding metadata. However, popular tools like sra-tools (https://github.com/ncbi/sra-tools), ffq, and fetchngs are limited to accession inputs (Ewels *et al.* 2020, Gálvez-Merchán *et al.* 2023). While programs like NCBI Entrez Direct (EDirect) and Entrez Programming Utilities (E-utilities) (Sayers *et al.* 2023) allow for keyword searches, they are not intuitive and require significant time to learn, as users must have programming knowledge or understand complex internal subfunctions. Moreover, even when specific data are retrieved, the metadata is typically provided in XML format, which is unfamiliar to most researchers. Processing this metadata into the experiment- and study-level table formats that Gencube offers demands substantial programming expertise and time.

**Figure 1.**
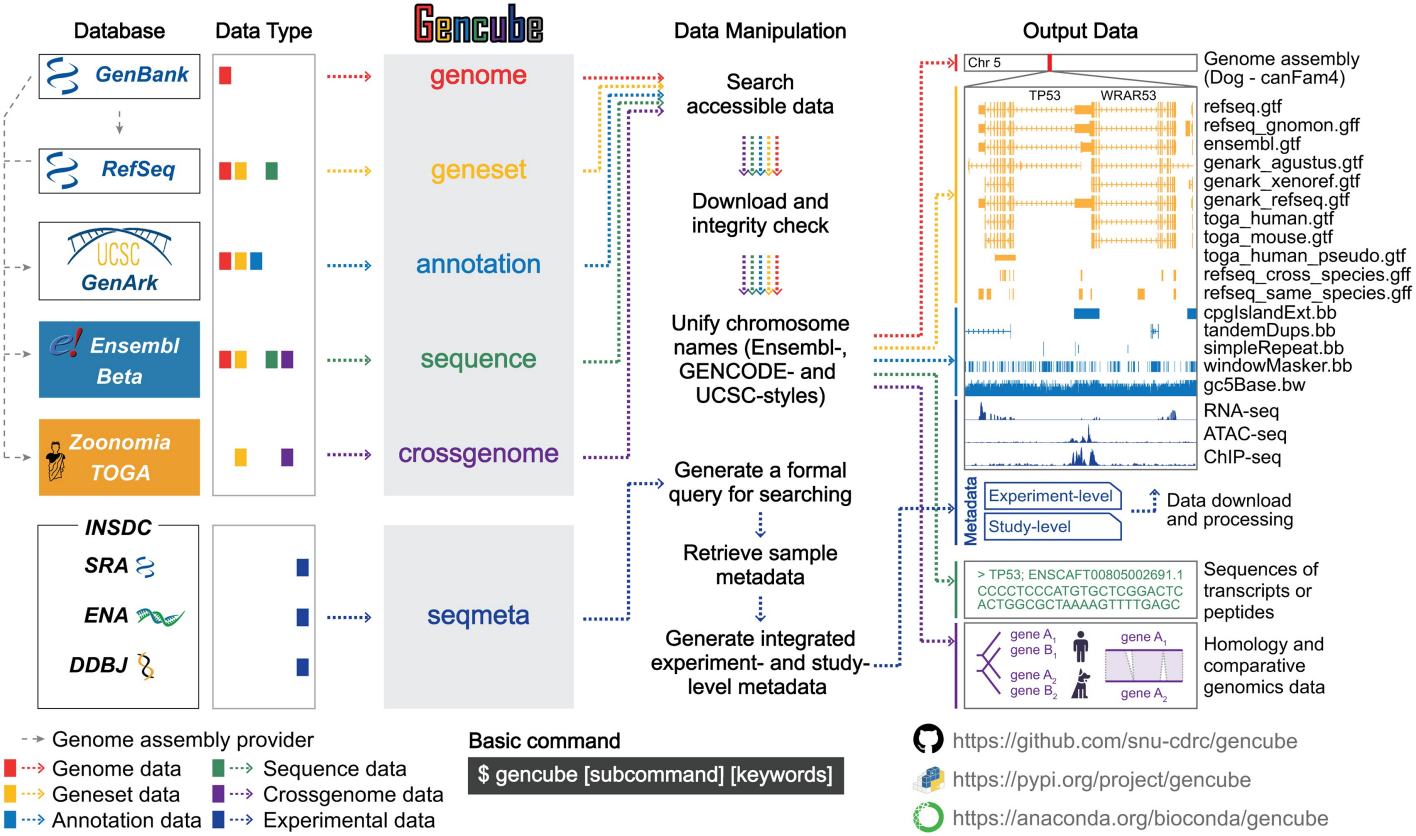
Overview of the Gencube workflow. The left panel lists the databases accessible through Gencube, along with the types of data retrieved. The middle panel illustrates the key subcommands and data manipulation processes. The right panel showcases examples of the output data that are downloaded and unified. The entire set of processes, from accessing the data to generating the final output, is color-coded according to the corresponding data type.

Here, we present Gencube, a Python-based, open-source command-line tool designed to streamline programmatic access to metadata and diverse types of multi-omics resources from publicly accessible leading repositories (Fig. 1). This software simplifies the retrieval and unification of genomic data, and facilitates the rapid exploration of high-throughput sequencing data, enabling researchers to efficiently collect datasets without resorting to labor-intensive and error-prone manual web methods. By enhancing data accessibility and reliability, Gencube empowers researchers to perform more accurate and effective multi-omics data analyses.

## 2 Description

Gencube consists of six key subcommands (Fig. 1), each dealing with different types of data:

- *gencube genome*: Fetches metadata and Fasta format files for genome assemblies.
- *gencube geneset*: Fetches GTF, GFF, or BED format files for gene annotations.
- *gencube annotation*: Fetches BigBed or BigWig format files for several types of genome annotations, such as gaps, GC percent, CpG islands, and repeats.
- *gencube sequence*: Fetches Fasta format files for transcript or peptide sequences.
- *gencube crossgenome*: Fetches comparative genomics data, such as homology or codon- and protein-alignment of genes from different species.
- *gencube seqmeta*: Searches for high-throughput sequencing data corresponding to user-specified keywords, retrieves the related sample metadata, and integrates it into experiment-level and study-level tables.

The first five subcommands collectively perform an initial search and fetch metadata of genome assemblies based on user requirements using NCBI E-utilities via Biopython (Cock *et al.* 2009). Researchers can use various forms of input accepted by NCBI Entrez, including scientific or common names of species, accessions, assembly names, and UCSC names, such as homo_sapiens, human, GCF_029378435.1, GRCh38, and hg38. After this initial step, each subcommand accesses one or several public databases to check the availability of the targeted data for the searched genomes and displays the results in the terminal. Except for GenArk and TOGA, repositories provide MD5 checksum information to ensure data integrity. If the checksum of a previously or newly downloaded file differs from the server’s checksum, the download will be retried once to maintain data consistency.

Although the sequence information of genomes is the same across databases, the genome subcommand allows downloads from multiple repositories because each database applies different masking methods. If masking affects the analysis, users can choose the appropriate source based on their needs and select between soft-masked, hard-masked, and unmasked genomes as required.

Consistent chromosome naming among the genome, gene set, and other annotations is crucial when analyzing data. This is because, during data processing, if the chromosome names in the files do not match, the annotation information cannot be correctly recognized, leading to errors or incomplete analyses. However, databases use various naming conventions, primarily falling into four categories: GenBank, RefSeq, Ensembl, and UCSC. According to user preferences, Gencube unifies chromosome names in files downloaded, converting them into Ensembl, GENCODE, or UCSC styles:

- Ensembl: Uses simple numeric and letter designations (e.g. 1, 2, X, MT). Unknowns use GenBank IDs.
- GENCODE: Uses “chr” prefix (e.g. chr1, chr2, chrX, chrM). Unknowns use GenBank IDs.
- UCSC: Also uses the “chr” prefix but employs UCSC-specific IDs for unknowns, with limited use if UCSC IDs are not issued.

**Figure 2.**
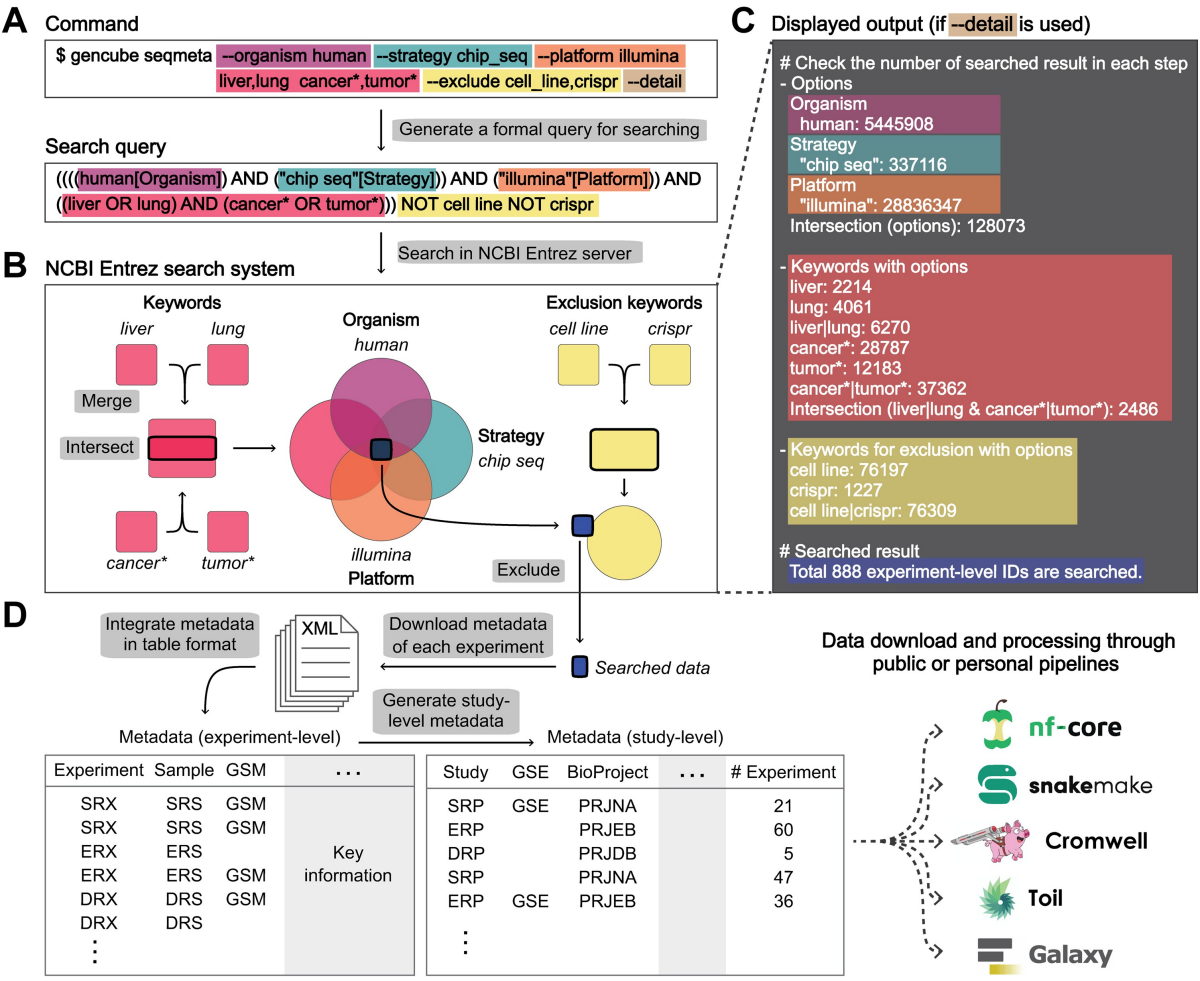
Schematic workflow of the seqmeta subcommand. (A) An example of the seqmeta subcommand and its converted form as a search query. (B) Synopsis of data retrieval for the search query from NCBI Entrez. (C) Displayed output related to the data retrieval step. Each part of the command and the corresponding part in panels B and C are marked with matched colors. (D) The seqmeta subcommand downloads experiment metadata for all search results, extracts core information, integrates experiment-level output into a table format, and finally generates study-level output.

The seqmeta subcommand also accesses sequencing data from the INSDC through NCBI Entrez. Initially, it generates a formal search query and implements all fields, properties, and filters available in Entrez as options (Fig. 2A). The input can include not only accession numbers but also a variety of relevant keywords. Additionally, wildcard (*) and the caret (^) at the end of the search terms can be used to include all entries containing the specified keyword (e.g. cancer* will include cancer, cancers, etc.) and perform exact word combination searches (e.g. liver_cancer^ will search for the exact phrase “liver cancer”).

Depending on how the command is structured, Boolean operators (AND, OR, NOT) are used to either broaden or narrow the scope of the search, ultimately outputting the final set of experiment IDs (Fig. 2B). By displaying the number of results based on different options and keywords at each step in the terminal, it helps in making decisions about the query composition during the search process (Fig. 2C). Finally, for all the obtained IDs, metadata in XML format are collected and converted into table format for intuitive understanding by the user, creating both integrated experiment-level and study-level outputs (Fig. 2D).

Gencube utilizes well-established packages to ensure robust server interaction and data processing capabilities. Specifically, it employs Pandas (The Pandas Development Team 2024) for efficient data manipulation, requests (https://requests.readthedocs.io) and BeautifulSoup4 (https://www.crummy.com/software/BeautifulSoup) for reliable web data access, and tqdm (https://github.com/tqdm/tqdm) for enhanced result presentation. The software has undergone comprehensive testing on Linux/Unix and Mac OS (Darwin) platforms, confirming its cross-platform functionality and stability.

To modify the BigBed and BigWig files (binary format) downloaded by the annotation subcommand, they need to be converted to BED and BedGraph formats in the chromosome name conversion step, respectively. After making the necessary modifications, these files are then restored to their original formats. In these processes, the UCSC genome browser utilities (bigBedToBed, bigWigToBedGraph, bedToBigBed, and bedGraphToBigWig) (Kent *et al.* 2010) are used instead of using a Python library.

## 3 Usage and documentation

Gencube can be installed via the command line using “pip install gencube.” Alternatively, it can also be set up with conda. Installation is extremely straightforward, and for researchers with even a basic understanding of the command-line interface, using Gencube is very easy. Users can invoke the help flag [-h] in the command line to receive detailed usage instructions. Additionally, a comprehensive, step-by-step manual, complete with examples is available on the GitHub page at https://github.com/snu-cdrc/gencube. This ensures that users have access to thorough guidance for all subcommand functionalities.

## 4 Discussion

The number of multi-omics resources submitted annually from various consortia and projects is increasing exponentially. In response, Gencube streamlines access by integrating multiple repositories into a unified command-line tool, eliminating the need for manual navigation and data parsing. Unlike existing tools, Gencube ensures consistency in data retrieval and format standardization, making it a valuable resource for a broad range of omics applications, including genomics, transcriptomics, and epigenomics. However, certain limitations currently exist.

First, Gencube accesses and downloads data from various repositories, and the speed can vary significantly depending on the repository selected. When using the seqmeta function, metadata is retrieved from the INSDC through APIs, and the rate limits imposed by NCBI can affect performance. Specifically, the absence or presence of an API key can lead to a more than three-fold difference in speed (3 requests per second without an API key; 10 requests per second with an API key). Therefore, we recommend that users register an API key when using Gencube to optimize retrieval speed.

Second, while there are numerous multi-omics data repositories, Gencube has focused on databases where programmatic access is currently unavailable or where essential additional functionalities are needed. We recognize this limitation and are committed to gradually expanding Gencube to include more databases, thereby enhancing its utility and providing greater value to users.

Lastly, although we have curated the most crucial information through the seqmeta function, the metadata submitted by researchers to INSDC is not yet perfectly standardized. Consequently, some manual selection and curation of experiments from the integrated metadata may be necessary for research use.

Conflict of interest: None declared.

## Data Availability

All data and code relevant to this article are available at: https://github.com/snu-cdrc/gencube.
